# Optimal Network of General Hospitals in Slovenia

**DOI:** 10.3390/ijerph20054235

**Published:** 2023-02-27

**Authors:** Patricia Blatnik, Štefan Bojnec

**Affiliations:** Faculty of Management, University of Primorska, Izolska Vrata 2, SI-6000 Koper, Slovenia

**Keywords:** network, general hospitals, accessibility, location–allocation, maximize attendance model, ArcGIS

## Abstract

This article explores how the minimum number of general hospital locations can be determined with optimal population coverage. Due to the increasing financial problems of hospitals and the poor organization of general hospital healthcare, Slovenia is currently working to reform the healthcare system. Defining the optimal network of hospital providers is one of the key elements in reforming the healthcare system. To define the optimal network of general hospitals, the allocation-location model was used, and the maximize attendance model was used as the central method. The chief purpose of maximize attendance model is to optimize the demand attendance with respect to distance and time spent getting to the point of demand. In the analysis of optimal locations and the number of Slovenian general hospitals, we used data on the locations of settlements with their number of inhabitants and data on the Slovenian road network, based on which we defined average travel speeds on the categorized road network. The hypothetical locations of general hospitals and the number of optimally located general hospitals that provide access to the nearest provider were determined in three different time intervals. We found that the same accessibility to hospital services as provided by the existing network of general hospitals can be achieved with only ten optimally located general hospitals within a 30-min time interval. This means that two general hospitals could be rationalized or reorganized, which would bring significant savings in the field of hospital activity, which creates a large loss in the health system in Slovenia.

## 1. Introduction

The organization of general hospital healthcare in Slovenia is half a century old. Demographics, infrastructure and the medical profession have changed significantly during this time, but the organization of general hospitals remains more or less the same, so redefining the general hospital network is a key issue. Ensuring an optimal health care network is also necessary to maintain and improve the general health status of the population [1,2]. In case when the working power of providers is not used in its full capacity or when providers have unsuitable locations, the health status of the entire population in the designed area is made substantially worse. Consequences of such a dangerous situation can be seen in the form of increased mortality and sickness of the population [3].

Due to the COVID-19 crisis, the accessibility of health services has come even more to the fore. General hospital accessibility is a key factor in identifying areas with healthcare shortages. This is also highlighted by an increasing number of authors who are engaged in research on accessibility to health care providers [4,5]. Optimization models enable exploration of the current access to the existing healthcare services, as well as to form new possible alternatives either with improved access to the existing providers of healthcare services, or with forming a completely new network of healthcare providers [6]. Access to healthcare services is usually determined for areas that have a regulated and organized access to the local road network [7,8]. The network of healthcare providers is influenced by numerous factors, such as area location, number of inhabitants, road network, geographical obstacles between supply and demand [9].

Several studies have shown that location–allocation models ensure scientific support during planning and defining of optimal locations of potential objects [10,11]. These models reduce the whole distance in question as well as time; this function renders it possible for the models to solve location problems and simultaneously determine the most effective locations for the objects in question; they also focus on those geographical areas that are most subjected to demand and have potential for a wide range of possibilities [10,12]. There are many different location–allocation models which differ with regard to problem-solving and mathematical operations [13,14].

Location–allocation models have been a case of study in numerous different fields ever since 1960. They were most frequently used in the fields of geography, economy, industry and public administration [15]. Many different classifications of these models can be observed in literature [16]. When conducting an analysis of optimal healthcare providers, the mentioned models in connection with the GIS were used, which means they are similar regarding contents and procedure. In most cases, we distinguish four different location–allocation models: minimal impedance model—MI-model, minimize facilities model—MF-model, maximize coverage model—MC-model, maximize attendance model—MA-model. 

The focus of the MI-model is that it directs itself to allocate a defined amount of healthcare providing facilities, in this case general hospitals, in a way that the average distance and time to reach them is shortened as much as possible for the inhabitants in need of healthcare services [6,17]. The MF-model looks for solutions how to utilize the minimal amount of available healthcare providers, that can still cover the requirements of inhabitants in need of healthcare services within the defined distance or time interval [18]. The MC-model attempts to maximize the coverage of the demand points, or in other words the coverage of the inhabitants, for which the healthcare service is available in that timespan or distance [6,17]. 

The MA-model attempts to maximize the attendance a provider has within the area of an appropriate time interval. Every point of demand does not hold the same weight; the larger the population, the greater is the demand [19,20]. Model MA requires a different ratio of demand and/or different weighted point of demand according to the distance covered by the patient when he attends the provider. Weight is reduced proportionally by increasing the distance between the provider and the patient [10,21]. The results of the MA-model are different from the other location–allocation models in the way that they define the optimal location for general hospitals in larger or more densely populated habitats. With this it can be ensured that newly placed general hospitals have maximum attendance, as they operate in the locations with the highest demand.

The purpose of this article is to present possible projections of network of hospitals in Slovenia, following the objective to form a new network within the vacant geographical area in Slovenia. A change in the organization of general hospital healthcare is necessary, as the current system is already 50 years old. The current distribution of general hospitals in the country is therefore as it corresponded to former transport links and social needs. If such an organization of general hospital distribution was once suitable for providing health care to residents, it is certainly not anymore today, so the medical and economic profession would arrange it much better and more rationally today, since we have a significantly better transport connection and thus better accessibility in the territory of the entire country [22].

The Ministry of Health reports on the large losses incurred by general hospitals in recent years. In the period 2017–2021 alone, Slovenian general hospitals show a cumulative loss of 93 million euro, which is why the Ministry of Health has repeatedly proposed to redefine the network of general hospitals and to merge or transform some general hospitals as part of measures to rehabilitate the business of health care providers [22,23]. The Slovenian Health Insurance Institute, which is the only public health insurance company in Slovenia and at the same time the payer of health services, also argues that Slovenian health care providers are too fragmented [22]. The Ministry of Health even prepared a legal regulation to facilitate the merging of public health institutions, but it has not yet been adopted [24].

Reorganization and merging of general hospitals should not take place only on the basis of their financial operations, but when defining the optimal network of general hospitals, geographical accessibility must be taken into account. Accordingly, in the article we present different projections of the optimal network of general hospitals that would be the most efficient considering the spatial population distribution according to the locations of settlements and road network development with estimated average travel speeds on the categorized road network. Such projections enable establishing new starting points of expertise regarding potential location changes of general hospitals or potential decisions on reducing the number of general hospitals. Locations of specific hospitals and the gravitation of the points of demand, especially when compared to the existing locations, offer a solid basis for future political decisions regarding health care. In accordance to this statement, the MA-model was chosen among the four location–allocation models, since it attempts to minimize the number of healthcare providers necessary to cover the maximum possible number of demand points based on the length of time needed for traveling.

## 2. Materials and Methods

### 2.1. Study Area

Our research explored possible solutions for defining the optimal network of general hospitals in Slovenia. Due to the increasing financial problems of general hospitals, the aging of the population, new road infrastructure, the placement of general hospitals in optimal locations is necessary. The healthcare system is being reformed in Slovenia, and one of the key issues is how to redefine the network of healthcare providers and at the same time maximize access to healthcare services for all residents. It is important to add that public transport in Slovenia is poorly organized, which is why 90% of the population still use private cars as a means of transport to access health services. The mentioned problems are also encountered in some other European countries, where they are also trying to redefine the network of healthcare providers and increase accessibility to general hospitals [25]. Accordingly, our aim was to analyze how to maximize accessibility to general hospital services, taking into account the locations of settlements with their number of inhabitants, data on the road network of Slovenia, on the basis of which we defined average travel speeds on the categorized road network. The sensitivity of the results is investigated with used different time intervals during which patients can access the nearest healthcare provider by private car. However, financial resources are an important category of accessibility to health services, but at the same time we must also consider geographical accessibility, which we focused on in the analysis. A similar approach was also used by some other authors who are engaged in research on access to health care [26].

### 2.2. Methodology

For the purpose of the chosen projection of optimal network of general hospitals the MA-model was used as the main method. The chosen model does not choose new locations of providers based on the already existing ones; it determines new locations completely independently of those already in existence.

The purpose of the MA-model is to determine new locations of providers, thus enabling them to maximize the attendance of patients. The model is able to find options for locating providers near the majority of the population [20]. In other words—optimal locations of providers in MA-models are going to be in direct proximity to the greatest number of points of demand.

The mathematical function used by this model was written down by Holmes, Williams and Brown [19]:

(1) $$Z=\sum_{i=0}^{n}\sum_{j=0}^{n}p_{i}\left(S-d_{ij}\right)x_{ij},$$

where, Z is the objective function, $p_{i}$ is the population of demand i, n is the demand points number, S is the threshold distance, $d_{ij}$ is the travel distance between i and j, and $x_{ij}=0$ if the demand point i is not covered by a facility j and $0<d_{ij}<1\;$ when the demand point i is sheltered.

**Figure 1:**
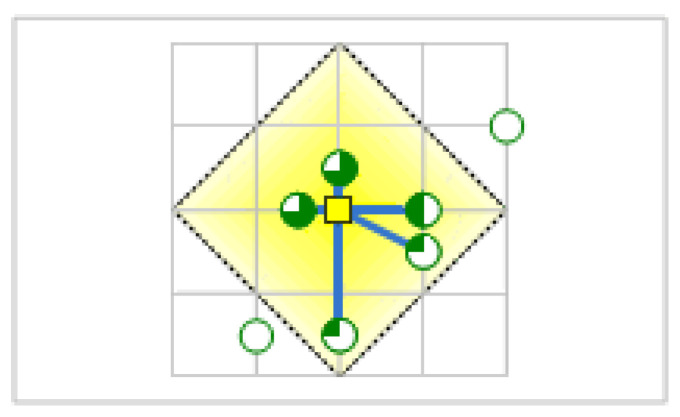
Display of point selection in the MA-model.

The MA-model chooses facilities such that as much demand weight as possible is allocated to facilities while assuming the demand weight decreases with distance. The demand points represented by pie charts in Figure 1 show how much of their total demand is captured by the facility.

The maximum time needed for traveling must be carefully determined so that providers can produce satisfactory clinical outcomes. There is no clearly defined opinion in literature or among the followers of healthcare policy on how to determine an appropriate time period for traveling to the nearest hospital; therefore, three different time intervals most frequently mentioned in theory were chosen. The decision on the length of the appropriate traveling time that would help formulate the optimal network of healthcare services should be made by those who engage in forming healthcare policy. The first time interval is defined as a 30-min long journey with a personal vehicle. The second time interval is defined as a 45-min long journey and the third time interval as a 60-min long journey. Consequently, three different solutions were obtained. These time intervals were selected based on Slovenian health policy documents and based on discussions in the media, appearing in conjunction with the new healthcare reform. These three different time intervals of accessibility are also the most frequently mentioned time intervals in theory [27].

The MA-model was chosen among other models encompassed by the location–allocation method and used it together with the ArcGIS (Network Analyst). GIS is also the most commonly used tool in studies of access to health services [28]. ArcGIS helped to prepare several geographical charts, which present the optimal hypothetical locations of hospitals that can be reached by patients (and with a personal vehicle) within the determined time intervals.

### 2.3. Data

During the analysis of optimal locations and the number of Slovene general hospitals, three different time intervals were used; simultaneously the information on locations of cities and villages was needed, along with the number of inhabitants, data on Slovene road network and information about location of cities with more than 5000 inhabitants. The official data regarding the location of cities and their total number of inhabitants were gained in cooperation with SORS (Statistical Office RS). SURS gathers information on the total number of inhabitants in each administrative unit and based on a hierarchical network of units equal in size. This basis enables to mark and connect any spatial unit with the gathered statistical data on the exact number of inhabitants within the given geographical chart. In order to define the exact number of inhabitants the spatial unit 500 m × 500 m was used. All the information is available to the public—the data from 2019 were used in this case.

Basic road network information was included in ArcGIS 10.9 and gathered based on the freely available digital map OpenStreetMap, a map used more and more frequently for the purpose of analyzing. OpenStreetMap was used because, unlike many other maps provided for this purpose, it is updated daily. The analysis required certain changes; therefore, it was remodeled and completed the information on road network gathered by OpenStreetMap. Categories unnecessary for the analysis—such as walking paths, bicycle routes, horse paths, bus stops—were eliminated as they were not relevant. However, several road sections OpenStreetMap did not provide were included. Next step was to enter the data on average travel speed into the road network. For this purpose, the official classification of state roads 2019 was used.

The number of inhabitants living within a settlement was received from SORS. This was supported by the objective, as it was desired to move locations of hospitals to those settlements that can boast at least 5000 inhabitants. The purpose is based on the fact that providers in densely populated areas can answer to a greater number of points of demand; simultaneously their opportunities for being frequently visited are much better than if they were located in sparsely populated areas. Based on the gathered data, 39 different settlements were determined.

## 3. Results

The MA-model weights measure demand points according to the distance from the user to the nearest contractor. The weighting decreases proportionally as the distance between the contractor and the demand point increases. This is consistent with the fact that in this model it was assumed that the possibility of visiting a certain provider decreases with increasing distance of the user to the nearest provider. Consequently, this means that demand is higher where the population is denser. Compared to other location–allocation models, the MA-model locates contractors in more densely populated areas.

**Figure 2:**
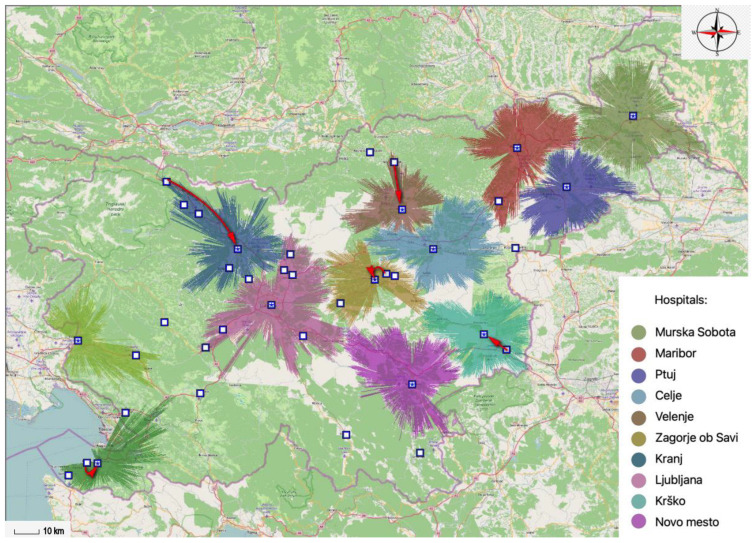
Comparison of existing and optimally located general hospitals within a 30-min time interval.

Within the existing network of general hospital providers, 12 general hospitals operate in Slovenia. Figure 2 shows the results offered by the MA-model looking for the optimal locations of twelve general hospitals within a 30-min time interval. If these results are compared with the locations of the existing twelve general hospitals, then the location of the Izola General Hospital is replaced by a location in Koper, the Jesenice General Hospital is newly located in Kranj, the Trbovlje General Hospital is moved to Zagorje Savi, the location of the Slovenj Gradec General Hospital is replaced by a location in Velenje, and the Brežice General Hospital is newly located in Krško. 

The proportion of the population covered by existing general hospitals and the proportion of the population covered by optimally located general hospitals are very similar. With the existing locations of general hospitals, it is covered 84.34% of the population and with optimally located general hospitals, it is covered 84.56% of the population within a 30-min time interval. In this case, the potential network of general hospitals could therefore provide health services to an additional 4627 inhabitants.

**Figure 3:**
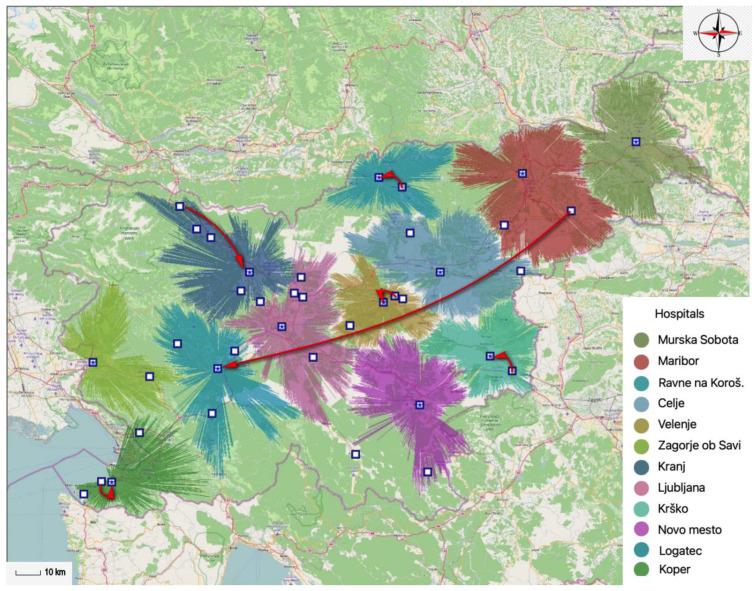
Comparison of existing and optimally located general hospitals within a 45-min time interval.

Similar results are obtained when comparing twelve existing general hospitals with twelve optimally located general hospitals within a 45-min time interval. In Figure 3, it can be seen that the location of the Izola General Hospital is replaced by a location in Koper, the Jesenice General Hospital is newly located in Kranj, the Trbovlje General Hospital is moved to Zagorje ob Savi, the location of the Slovenj Gradec General Hospital is replaced by a location in Ravne na Koroškem, while the Brežice General Hospital is newly located in Krško. It should also be pointed out that within the model, on the one hand, the Ptuj General Hospital has been discontinued, but on the other hand, the model has redefined the general hospital, which should be located in Logatec.

The proportion of the population covered by existing general hospitals is slightly lower compared to the proportion of the population covered by optimally located general hospitals. With the existing locations of general hospitals, it is covered 95.36% of the population and with optimally located general hospitals, it is covered 96.91% of the population within a 45-min time interval. In this case, the potential network of general hospitals could therefore provide health services to an additional 31,949 inhabitants.

The MA-model has proven to be the most effective model for determining the optimal network of general hospitals. Based on this model, it is easiest to follow the goal of defining optimal locations and the number of general hospital providers, on the assumption of finding a balance between maximizing the economic efficiency of health care providers and maximizing accessibility to health services.

On one side, maximization of the accessibility of general hospitals providers was attempted, but on the other side acknowledgment that healthcare system comes with limited funds at the disposal. Therefore, while searching for locations and determining the number of general hospitals, it needs to be considered that general hospitals cannot be located in a way that would cover all basic needs of the population. It would only maximize accessibility, while simultaneously neglecting the fact of limited funds. If the matter is viewed in such light, it must acknowledge that finding a specific number of general hospitals, which will serve the greatest possible capacity of the population with attendance to healthcare providers, is the most suitable solution for forming the optimal network of general hospitals. Our analysis also provides an answer to the question of which general hospitals in Slovenia would make sense to merge and reorganize. The minimum number of general hospitals within chosen time intervals was determined on a coverage rate of 80% of the population. This is a similar percentage of coverage as the existing network of general hospitals, but at the same time it is also the average coverage rate that serves as a standard in the majority of countries in the world [29].

The MA-model indicates only ten optimally located general hospitals are needed within the 30-min time interval. This way general hospital services are available to 80.74% of the total population within the allotted 30-min time interval. In accordance with this statement, 1,665,488 inhabitants of Slovenia would have access to a general hospital within the 30-min time interval. By reducing the number of general hospitals by one, the coverage rate is still 78.11% of the population. With nine optimally located hospitals it is therefore ensured access to general hospital services providers for 1,611,285 inhabitants of Slovenia. The gathered data on the number of general hospital and the percentage of the population gravitating toward a particular general hospital location within the 30-min time interval are displayed in Table 1.

**Figure 4:**
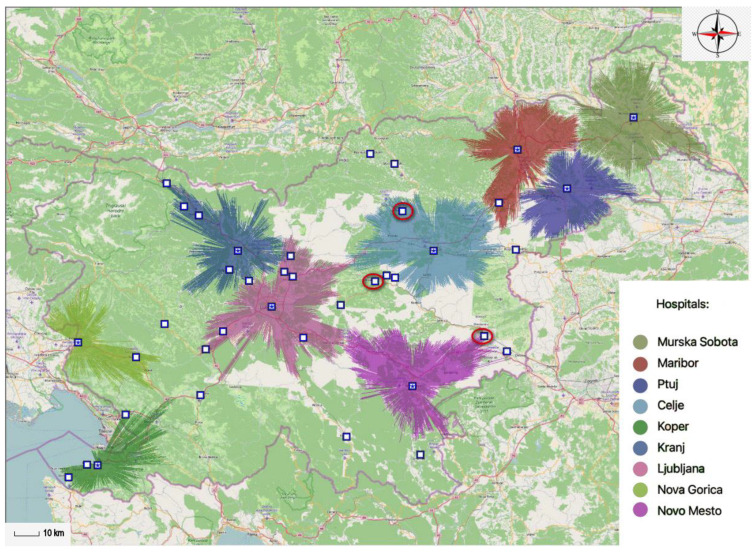
Optimal locations of nine different general hospitals within the 30-min time interval.

**Figure 5:**
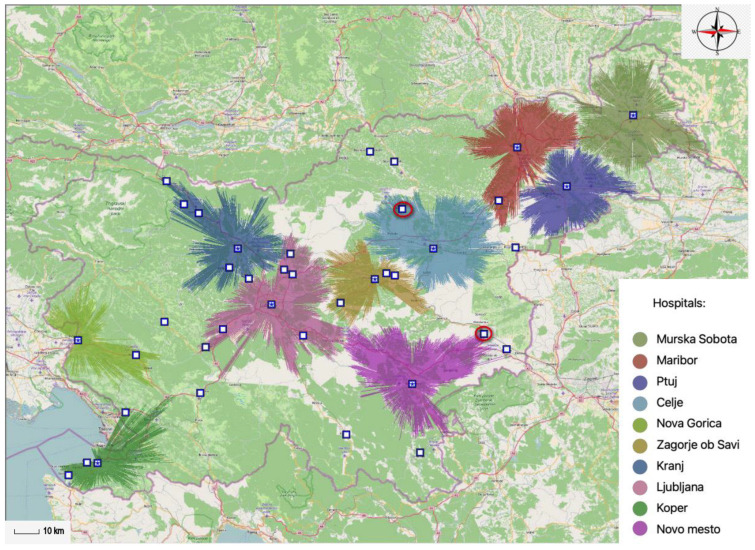
Optimal locations of ten different general hospitals within the 30-min time interval.

Figure 4 and Figure 5 display the results acquired by the MA-model. The figures show two projections of spatial accessibility of the optimally located general hospitals within the 30-min time interval. Figure 4 displays nine different optimal locations of Slovene general hospitals within the 30-min time interval, covering 78.11% of the total population. In this case, the MA-model excludes the general hospital that was initially located in Velenje, the general hospital that was in Zagorje ob Sava, and the general hospital that was in Krško.

Figure 5 displays ten optimal locations of Slovene general hospitals within the 30-min time interval, covering 80.74% of the total population. In this case, the criteria for the 80% coverage rate of the population were met, which was essentially the main objective. The results show us that the general hospital, which was located in Velenje in the initial state within the MA-model, and the general hospital, which was located in Krško, are exempt.

Within the 45-min time limit five general hospitals need to be allocated. This way general hospital services are available to 84.35% of the total population within the 45-min time interval. In accordance with this statement, 1,740,072 inhabitants of Slovenia would be able to reach the nearest hospital within the 45-min time interval. By reducing the number of general hospitals by one, the coverage rate is reduced to 77.65% of the population. Thus ensuring 1,601,793 inhabitants are able to access general hospital services within the 45-min time interval with only four general hospitals. The data are displayed in Table 2—it illustrates the data on the number of general hospitals and the percentage of the population, gravitating toward any particular optimal general hospital location within the 45-min time interval.

**Figure 6:**
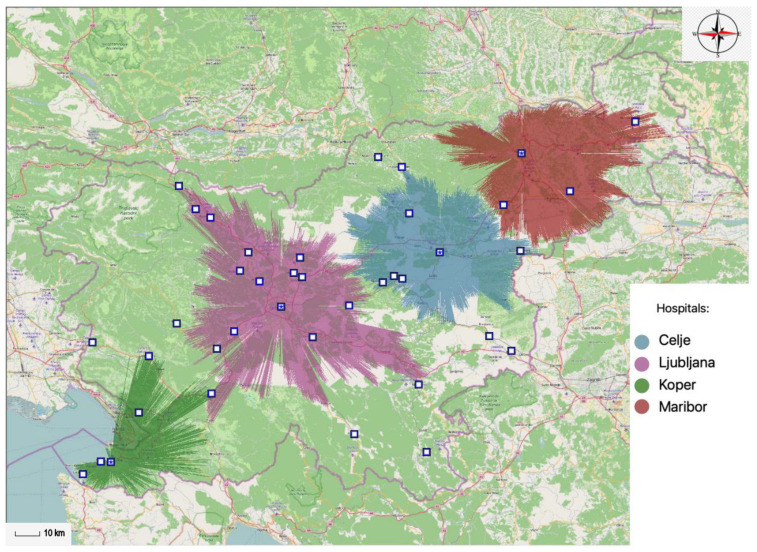
Optimal locations of four different general hospitals within the 45-min time interval.

**Figure 7:**
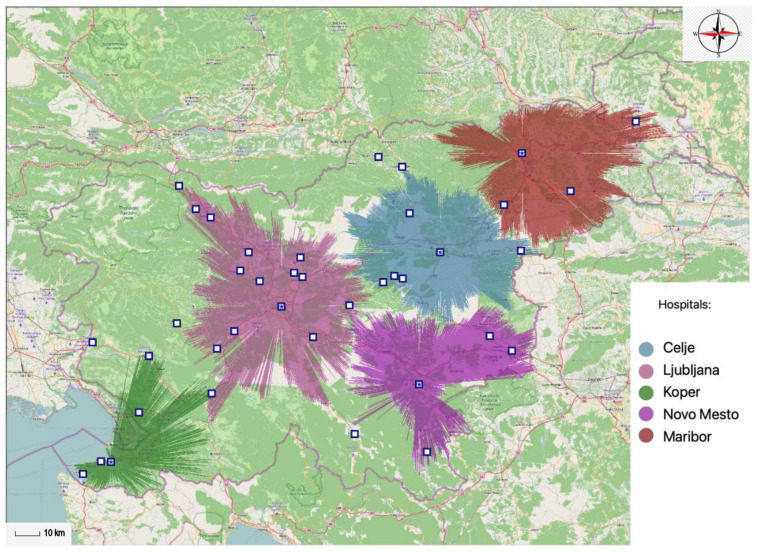
Optimal locations of five different general hospitals within the 45-min time interval.

Next the projections of spatial accessibility of optimally located general hospitals within the 45-min time interval are displayed. Figure 6 and Figure 7 show the results supplied by the MA-model; by illustrating the optimal locations of general hospitals. Figure 6 shows four optimal locations of Slovene general hospitals within the 45-min time interval, which together cover 77.65% of the population.

Figure 7 displays five optimal locations of Slovene general hospitals within the 45-min time interval, thus covering 84.35% of the population. In such case the general coverage rate standard is exceeded, which bodes well for greater accessibility; it also offers benefits to the patients that require medical care.

By increasing the interval of accessibility to a 60-min long journey, the number of general hospitals necessary declines drastically. Only two general hospitals are needed to satisfy attendance within the 60-min time interval. In such case healthcare services are available to 81.45% of the population within the 60-min time interval and include 1,680,207 inhabitants of Slovenia. By reducing the number to only one (hospital), the coverage rate decreases to 55.49% of the population; in terms of building the optimal network of general hospital services, the percentage is too low. In such case (only one general hospital available) only 1,144,725 inhabitants of Slovenia would have access to the hospital within the 60-min time interval. This is best illustrated with Table 3; it displays the data on the number of general hospitals and the percentage of the population, gravitating toward a specific optimal general hospital location within the 60-min interval.

**Figure 8:**
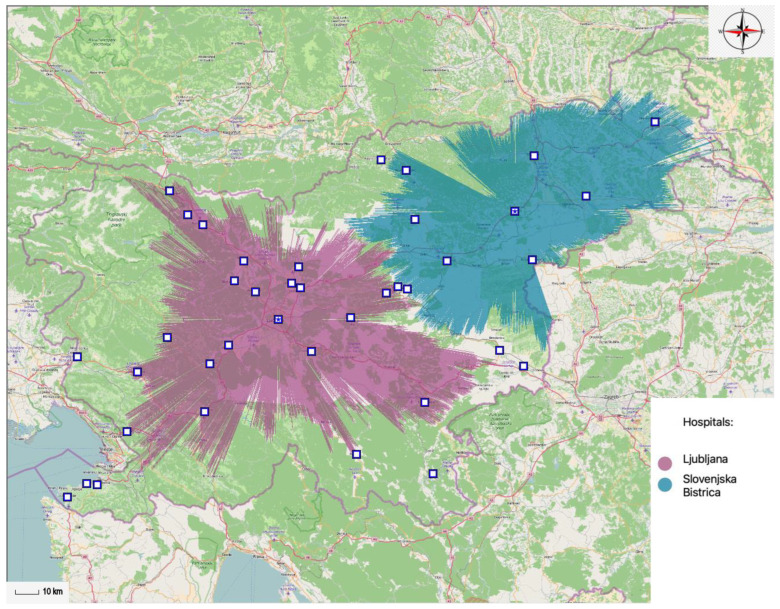
Optimal locations of two different general hospitals within the 60-min time interval.

Figure 8 below this section displays the results gathered by the MA-model. The projection of spatial accessibility is illustrated with only two optimally located general hospitals. Figure 8 shows the network of optimally located general hospitals within the 60-min time interval, covering 81.45% of the population.

For comparison potential network with the current network Table 4 shows representation of the number of inhabitants and the percentage of population inside three time intervals which tend to specific general hospital of the existing network.

## 4. Discussion

The key contribution of this article is the formation of a model of the network of general hospitals, which enables to explain the factors of the healthcare network and is based on the theory of location–allocation. With such an outlook, the main objective of this study is fulfilled, which is to form and test the general hospital network model in Slovenia. To answer the many questions about the healthcare network is essential for every healthcare system in the world, not only in Slovenia, as they do not only define supply site in the field of healthcare, they are also crucial when it comes to rational query about the demand of healthcare services. The healthcare network around the world and in literature is known as one of the key nonfinancial mechanisms of selection and with that also of rational behavior of patients, as they are the users of healthcare services. Maximizing access to health services must be one of the key measures also within the framework of the new health reform that is being prepared in Slovenia this year. Better health of the population cannot be achieved without greater accessibility of health services. This has already been pointed out by some other authors in their studies [30].

Several geographical charts were prepared, illustrating the optimal hypothetical locations of general hospitals that are accessible to patients with a personal vehicle within three different time intervals. The decision on the acceptable time interval that would serve as a standard for forming the network of healthcare is an open question for healthcare policy. The first time interval of accessibility was defined as a 30-min long journey with a personal vehicle, the second time interval as a 45-min long journey and the third time interval as a 60-min long journey. Consequently, three different conclusions were obtained about the hypothetical locations of optimally located general hospitals and about the hypothetical number of general hospitals that can still provide access to healthcare institutions for the 80% of population.

In accordance with the results of the analysis of projections of optimal network of general hospitals, the standard of maximum accessibility interval still acceptable to a patient was defined; on this basis the optimal locations of Slovene general hospitals were determined. The areas with more than 5000 inhabitants as the potential locations were taken into account. Similar criteria were used by numerous prominent authors who worked on analyses of spatial accessibility of healthcare services [31,32]. Results usually vary depending on the prescribed criteria; in this study emphasis was done on stress on how optimal locations and the number of necessary general hospitals change according to the variation of the chosen time interval. This is one of the key factors in most studies that focus on defining optimal locations of healthcare providers [32,33,34]. With different models, defining the maximum time of travel, several different forms of network and several possible locations of general hospital on Slovenian grounds was shown.

The majority of authors built their studies/research on the belief that the most appropriate traveling time a patient needs to reach the nearest hospital is the chosen 30-min time interval [35,36]. In this case the results show it can be guaranteed the coverage rate of 80% of the population with ten optimally located hospitals. The optimal locations of general hospitals would be located within the network in the following cities: Celje, Murska Sobota, Ljubljana, Kranj, Ptuj, Maribor, Nova Gorica, Koper, Zagorje ob Savi and Novo mesto. In comparison to the existing state of affairs, the number of providers would be reduced by two hospitals. This means that it would be necessary to reorganize the Brežice General Hospital and the Slovenj Gradec General Hospital, which are no longer needed in the proposed new network. In addition, some general hospitals would be relocated. Jesenice General Hospital would be relocated to Kranj and Trbovlje General Hospital would be relocated to Zagorje ob Savi.

In Slovenia, within the framework of the health care system, we are facing the problem that we do not have organized nursing hospitals that would be fully operational, as dictated by the needs of patients. Accordingly, our proposal would be that the existing general hospitals, which are no longer needed in the new network, should not be closed, but transformed into nursing hospitals.

Several authors [37] state that the 45-min time interval is actually the most convenient one. In accordance with the longer time interval of accessibility, Slovenia would need five general hospitals. The optimally located hospitals would be situated in five cities: Celje, Ljubljana, Koper, Maribor and Novo mesto. According to existing network, it would require seven less general hospitals within the potential network and Izola General Hospital would be relocated to Koper.

Brabyn and Skelly [38] state it is acceptable to provide patients with medical attention within the 60-min time interval. However, they also warn that the medical condition of those patients that need an hour to reach the nearest hospital could be compromised. In this case Slovenia would require only two general hospitals. Two optimally located general hospitals would be in Ljubljana and in Slovenska Bistrica.

As in the existing network of general hospitals providers, also in the potential network, some geographical areas stand out and do not have access to the general hospitals inside a 30-, 45- and 60-min time interval. Their travel time of accessibility is prolonged. The solution to such areas can be searched by allocating private providers of healthcare services, which is of course a matter of agreement and up to decision makers in healthcare. At this point it is necessary to consider the outcomes for patients, but in the present study this was not measured. This should be done in agreement with the medical profession and decisions makers in healthcare.

Due to the aging population, increasing public awareness and especially due to the introduction of modern medical technology, the healthcare system is gradually facing the need to spend a greater amount of resources. This represents a very severe problem, because the already existing general hospitals in Slovenia generate losses of several million euro every year, which is why the need for rationalization has arisen. In doing so, we must not forget that it is also necessary to maintain or even increase accessibility to health services, which has deteriorated considerably during the COVID crisis [39]. It is expected that the pressure of increased medical costs would play a major role in the future, thus acknowledging the necessity for a rational management of costs [40]. Meden-Vrtovec and other authors [41] thus presented a feasible case of rationalization in terms of reducing healthcare services in case of maternity hospitals in Slovenia. 

The Ministry of Health has repeatedly proposed the closure of the Kranj Gynecology and Obstetrics Hospital and some other hospitals, but it has met with negative resistance [22,24]. According to the results of our study, it would make sense to transform the Kranj Gynecology and Obstetrics Hospital into a general hospital, while the other general hospitals that are no longer needed in the new network would be transformed into nursing hospitals. The projections shown in this study are the first such case in Slovenia, professionally analyzing possibilities of rational management in the field of healthcare.

The main limitations of the study are certainly the fact that the abolition of certain general hospitals and the establishment of new ones would require political consent at the national level. The need to reorganize general hospital healthcare is widely known in Slovenia, which is why our general hospital network projections are an excellent tool for decision makers. Of course, rationalization itself will be difficult to carry out in practice, as resistance is expected from many stakeholders in healthcare.

## 5. Conclusions

The analysis is presented with two conclusions. The first defines the hypothetical optimal locations of hospitals that can be reached by a patient within the three stated time intervals. The second conclusion offers the number of optimal locations, a hypothetical number of general hospitals, which would ensure constant healthcare for 80% of the population in Slovenia within the stated time intervals.

If healthcare policy makers decide all general hospital services should be accessible to patients within the 30-min time interval, there is a need of ten general hospitals in Slovenia. If they chose the 45-min time interval of accessibility, there is a need of five general hospitals. However, if 60-min time interval is chosen than Slovenia needs to have only two general hospitals. It is necessary, however, to bear in mind that potential hospitals should be of different sizes depending on specific solution. Their size should also differ accordance with the percentage of the population that gravitates toward a particular hospital. This deduction brings a new study and presents new starting points for political discussion.

If we want to achieve the goal of the health care network, which is to ensure that each individual health service is geographically as close as possible to each individual resident in the form that medical technology makes possible, then it makes sense to use the spatial accessibility projection model, which ensures accessibility to the nearest health service provider to the eighty percentage of the population within a 30-min time interval. In this way, we ensure maximum benefit for the individual requesting health services, as the mentioned spatial accessibility projection model covers the shortest time interval that patients need to reach the nearest health service provider. At the same time, with this model, taking into account the optimal size or the optimal number of medical services that general hospitals should provide, we ensure that the providers cover the population’s needs for hospital services. Within the selected optimal model of the secondary health activity network, the providers would perform approximately the same number of health services as all general hospitals within the existing secondary health activity network. With ten general hospitals, which annually perform from 245,831 to 274,992 patient treatments, we thus provide approximately the same number of medical services as with the existing twelve general hospitals. 

The results of our research are useful in the light of the new healthcare reform that is being prepared to reform the healthcare system in Slovenia. In the context of the political debate, the key question is how to reform high loss-making hospitals without reducing access to health services. Our analysis offers an answer to the mentioned problem, as we have shown that with ten hospitals, we can provide almost the same accessibility to health services as with the existing twelve.

## Figures and Tables

**Table 1:** Number of general hospitals and a coverage rate of the population within the 30-min time interval.

Hospital	Nine Optimally Located Hospitals		10 Optimally Located Hospitals	
	Number of Inhabitants	Percentage of Population (%)	Number of Inhabitants	Percentage of Population (%)
Celje	220,370	10.68	209,607	10.16
Murska Sobota	108,637	5.27	108,637	5.27
Ljubljana	482,380	23.38	479,671	23.25
Kranj	205,165	9.95	205,165	9.95
Ptuj	84,113	4.08	84,113	4.08
Maribor	218,664	10.60	218,664	10.60
Nova Gorica	81,453	3.95	81,453	3.95
Koper	101,980	4.94	101,980	4.94
Zagorje ob Savi	/	/	67,710	3.28
Novo mesto	108,523	5.26	108,488	5.26
Total	1,611,285	78.11	1,665,488	80.74

**Table 2:** Number of general hospitals and a coverage rate of the population within the 45-min time interval.

Hospital	Four Optimally Located Hospitals		Five Optimally Located Hospitals	
	Number of Inhabitants	Percentage of Population (%)	Number of Inhabitants	Percentage of Population (%)
Celje	315,598	15.30	314,736	15.26
Ljubljana	766,845	37.17	736,901	35.72
Maribor	380,107	18.43	380,107	18.43
Koper	139,243	6.75	139,243	6.75
Novo mesto	/	/	169,085	8.20
Total	1,601,793	77.65	1,740,072	84.35

**Table 3:** Number of general hospitals and a coverage rate of the population within the 60-min time interval.

Hospital	One Optimally Located Hospital		Two Optimally Located Hospitals	
	Number of Inhabitants	Percentage of Population (%)	Number of Inhabitants	Percentage of Population (%)
Ljubljana	1,144,725	55.49	973,523	47.19
Slovenska Bistrica	/	/	706,684	34.26
Total	1,144,725	55.49	1,680,207	81.45

**Table 4:** Distribution of population across existing general hospitals.

Hospital	To 30 min Time Interval		From 30 to 45 min Time Interval		From 45 to 60 min Time Interval	
	Number of Inhabitants	Percentage of Population	Number of Inhabitants	Percentage of Population	Number of Inhabitants	Percentage of Population
/	323,092	15.66	95,752	4.64	24,310	1.18
Brežice	55,442	2.69	67,462	3.27	67,462	3.27
Celje	208,611	10.11	241,756	11.72	244,662	11.86
Izola	92,606	4.49	106,614	5.17	117,118	5.68
Jesenice	85,143	4.13	93,632	4.54	93,977	4.56
Ljubljana	588,629	28.53	662,164	32.10	693,748	33.63
Maribor	219,227	10.63	228,290	11.07	228,889	11.10
Murska Sobota	108,559	5.26	119,169	5.78	119,169	5.78
Nova gorica	81,453	3.95	100,893	4.89	107,428	5.21
Novo mesto	92,203	4.47	115,743	5.61	133,484	6.47
Ptuj	84,107	4.08	93,383	4.53	93,459	4.53
Slovenj Gradec	60,552	2.94	68,331	3.31	69,085	3.35
Trbovlje	63,250	3.07	69,685	3.38	70,083	3.40
Total	2,062,874	100.00	2,062,874	100.00	2,062,874	100.00

## Data Availability

Not applicable.
